# HOW DOES THE USE OF HIGH-COST TREATMENTS IMPACT UTILIZATION OF THE MEDICARE HOSPICE BENEFIT?

**DOI:** 10.1093/geroni/igae098.0750

**Published:** 2024-12-31

**Authors:** Michael Plotzke, Thomas Christian

**Affiliations:** Abt Associates, St. Louis, Missouri, United States; Abt Associates, Cambridge, Massachusetts, United States

## Abstract

Medicare patients who may benefit from palliative chemotherapy, radiation, or blood transfusions for symptom management (hereafter called “high-cost treatments”) may face difficulties accessing those treatments while electing hospice. The Medicare hospice benefit allows these high-cost treatments, but the treatments’ cost may exceed hospice agencies’ Medicare payments, limiting hospices’ ability to cover high-cost treatments. Lack of access to high-cost treatments may discourage transitions to hospice, resulting in costly and less patient-centered care. Using 100% Fee-for-Service (FFS) Medicare Part A/B claims, we examine how frequently these high-cost treatments are used at the end of life and how hospice use varies by the receipt of high-cost treatments. 7,631,620 FFS Medicare beneficiaries died from FY 2017-2021 and roughly 19.8% of those received at least one high-cost treatment in the 180 days before death (8.3% used chemotherapy, 3.73% used radiation, 12.8% used transfusions) and 47.1% used hospice in the 180 days before death. Of the 786,402 beneficiaries that used both hospice and at least one high-cost treatment in the 180 days before death, only 16,478 (2.1%) used hospice and a high-cost treatment simultaneously. We also found that the average hospice length of stay for those using high-cost treatments in the last 180 days of life is substantially shorter than for beneficiaries electing hospice without using those services (chemotherapy recipients - 22.9 days, radiation recipients – 23.2 days, transfusion recipients -25.5 days, non-recipients – 105.6 days). These findings may indicate barriers to receiving hospice for beneficiaries who may benefit from high-cost palliative treatments.

